# A quantitative assay for the assessment of cutaneous human papillomaviruses and polyomaviruses over time: A proof-of-concept in two patients with atopic dermatitis and psoriasis

**DOI:** 10.1371/journal.pone.0297907

**Published:** 2024-04-03

**Authors:** Aurélie Du-Thanh, Vincent Foulongne, Olivier Dereure, Marc Eloit, Philippe Pérot

**Affiliations:** 1 Pathogen Discovery Laboratory, Institut Pasteur, Paris, France; 2 Pathogenesis and Control of Chronic Infections, INSERM, University of Montpellier, Montpellier, France; 3 Département de Dermatologie, CHU de Montpellier, Montpellier, France; 4 Laboratoire de Virologie, CHU de Montpellier, Montpellier, France; 5 Ecole Nationale Vétérinaire d’Alfort, Maisons-Alfort, France; Korea Disease Control and Prevention Agency, REPUBLIC OF KOREA

## Abstract

The human skin virome, unlike commensal bacteria, is an under investigated component of the human skin microbiome. We developed a sensitive, quantitative assay to detect cutaneous human resident papillomaviruses (HPV) and polyomaviruses (HPyV) and we first used it to describe these viral populations at the skin surface of two patients with atopic dermatitis (AD) and psoriasis (PSO). We performed skin swabs on lesional and non-lesional skin in one AD and one PSO patient at M0, M1 and M3. After extraction, DNA was amplified using an original multiplex PCR technique before high throughput sequencing (HTS) of the amplicons (named AmpliSeq-HTS). Quantitative results were ultimately compared with monoplex quantitative PCRs (qPCRs) for previously detected viruses and were significantly correlated (R^2^ = 0.95, ρ = 0.75). Fifteen and 13 HPV types (mainly gamma and beta-HPVs) or HPyV species (mainly Merkel Cell Polyomavirus (MCPyV)) were detected on the skin of the AD and PSO patients, respectively. In both patients, the composition of the viral flora was variable across body sites but remained stable over time in non-lesional skin samples, mostly colonized with gamma-papillomaviruses. In lesional skin samples, beta-papillomaviruses and MCPyV were the major components of a viral flora more prone to vary over time especially with treatment and subsequent clinical improvement. We believe this method might be further used in extensive studies to further enhance the concept of an individual cutaneous viral fingerprint and the putative role of its alterations through various skin diseases and their treatments.

## Introduction

Although extensively studied using culture-independent methods, the description of the skin microbiome is mainly restricted to bacteria and fungi or parasites [1]. The human virome is an underestimated component of the human microbiome, mostly because of technical issues to characterize viral communities, lacking consensus sequences such as 16S rRNA or 18S rRNA and requiring less affordable whole genome sequencing (WGS) techniques and subsequent biostatistical analyses. Nevertheless, evidence that viruses can be commensal in the skin is both ancient and increasing. Nonpathogenic beta and gamma Human Papillomaviruses (HPV) are acquired early in infancy in the superficial layers of the skin [2]; the diversity of the skin virome is very high at both intra and inter-individual levels, as previously described [3–5]. Viruses detected on healthy skin samples are dominated by HPVs, human polyomaviruses (HPyV) like Merkel Cell polyomavirus (MCPyV), circoviruses, but also bacteriophages inhabiting P. acnes and Staphylococcus. Quantitatively, HPVs and HPyVs are the most important representants of viruses on the human skin surface [3]. For all these authors, the inter-individual variability of viral populations was high enough to lead to the notion of “unique viral fingerprint” [4].

HPVs and HPyVs are long-term resident viruses tolerated by the immune system that may promote skin healing [6]. Therefore, significant modifications of the skin virome might be associated with inflammatory reactions or with other pathogenic virus infections [7].

In genetically immunocompromised patients, commensal gamma-HPV usually shed by healthy individuals may be responsible for florid warts [8].

Here we describe the use of a quantitative assay to assess the HPV and HPyV populations over time in various skin sites of two individuals suffering from atopic dermatitis (AD) and psoriasis (PSO).

## Materials and methods

### Patients

Patient 1 was a 54-year-old atopic man suffering severe AD since childhood, refractory to superpotent topical steroids (STS), topical tacrolimus and phototherapy. Skin swab samples were taken from lesional skin (LS) of the ante cubital fossae and from non-lesional skin (NLS) of the inguinal folds before treatment (M0, June 2015), and at 1 (M1, July 2015) and 3 (M3, September 2015) months after an immunosuppressive therapy with methotrexate (MTX) 15mg/week was introduced because on-label use of cyclosporin A was contra-indicated by hypertension.

Patient 2 was a 66-year-old woman suffering moderate to severe cutaneous psoriasis (PSO) since 25 years, intermittently treated with an association of STS and topical vitamin D. Skin swab samples were taken from LS of both elbows and from NLS of the inguinal folds before treatment (M0, June 2015), and at 1 (M1, July 2015) and 3 (M3, September 2015) months after STS were applied regularly once a day.

Neither of the patients had received anti-HPV vaccination, nor had used STS during the month before sampling. Over time, the lesions of AD and PSO improved with treatment. Neither of them used topical emollients. According to ICH-GCP (https://database.ich.org/sites/default/files/E6_R2_Addendum.pdf), a written informed consent was obtained for both patients. This study was approved by Ethical committee (approval number 2014/38NICB), CCTIRS (approval number 15.699), CNIL (decision DR-2016-275), and ABM (approval number PFS-14-013).

### Samples

According to the recommendations by Kong et al. as regard the skin sampling [9], we used dry swabs thoroughly rubbed on LS (bilateral antecubital fossae in patient AD, bilateral elbows in patient PSO) and on the NLS of the bilateral inguinal folds for both patients. Sampling was done in the morning while the patients had not had a shower/bath since at least 12

hours. Skin swabs were immediately placed in PBS solution, which was then 0.2 nm-filtered, frozen and stored at -80°C, and identified individually.

### DNA extraction from skin swabs

DNA extraction was performed within 24 hours using a QiAmp DNA Mini Kit (QIAGEN, Courtaboeuf, France) according to manufacturer’s instructions. DNA extracts were stored at -80°C before proceeding to HTS.

### Ampliseq custom panel

We made a selection of 202 reference HPV and HPyV genomes according to the 2010 classification of papillomaviruses [10], this selection being performed before misclassifications were identified in 2021 in the HPV Gene Data Bank [11]. In detail, 64 alpha-, 47 beta-, 75 gamma- and 3 Mu-Nu-HPV, plus 13 known HPyVs [12], were selected. An Ion-AmpliSeq custom DNA panel was designed using the Ion AmpliSeq Designer (Life Technologies, Carlsbad, CA, USA), with an average amplicon size of 200bp. Briefly, a rough set of primer pairs was first generated to meet PCR thermodynamic and specificity criteria, leading to 1–32 candidate primer pairs per genome. A second-round selection was made to reduce the risk of inter-genomes (virus specificity) and intra-genomes (long-range amplification) cross reactivity. Finally, only 2 primer pairs per genome were kept, except for 4 viruses (HPV75, HPV143, HPV151 and HPV171) for which only 1 primer pair was kept due to design constraints. The final Ampliseq custom panel is made of a pool of 800 equimolar primers (400 primer pairs) and is registered under ID WG00164_2.

### NGS libraries and sequencing

After extraction of total DNA from skin swabs, the targeted DNA sequences were amplified with custom panel WG00164_2. Barcoded adapters were then ligated to the amplicons and the amplified barcoded library was purified and quantified using the Agilent 2100 Bioanalyzer instrument. The barcoded library was transferred to Ion Sphere Particles (Life Technologies, Carlsbad, CA, USA) to perform an emulsion PCR and enrichment of the Ion Sphere Particles according to manufacturer’s instructions. The library was further sequenced on the Torrent PGM System (Life Technologies, Carlsbad, CA, USA) on a P1 chip v2, following the manufacturer’s instructions.

### Bioinformatics analysis

Raw reads were trimmed to remove low quality bases and sequencing adapters at their ends with AlienTrimmer [13] (version 0.4.0, options -k 10 -m 5 -l 50 -p 80 -q 20) and mapped against the reference viral database of 202 genomes and the human genome (hg19) using Bowtie2 [14] (v2.2.6 with default parameters). Read counts are described for each patient in S1 and S2 Tables, respectively.

### qPCR

Relationship between read counts and quantitative monoplex PCR assays were conducted for six of the most represented viruses (HPV 107, 50, 134, 120, 12, and MCPyV) using the same primer pairs, individually, than those present in the AmpliSeq pool (S3 Table). Plasmids containing the corresponding amplicons sequences (S3 Table) were used as positive controls and quantification of the viral loads was made with standard curves generated by 10-fold dilution of the corresponding plasmids. Each qPCR was done in duplicate and the mean of the duplicate values was used for the correlation graph, null values were further excluded (S1 Fig).

## Results

### Detection and quantification of the cutaneous human papillomavirus and polyomavirus flora by AmpliSeq-HTS

**Fig 1:**
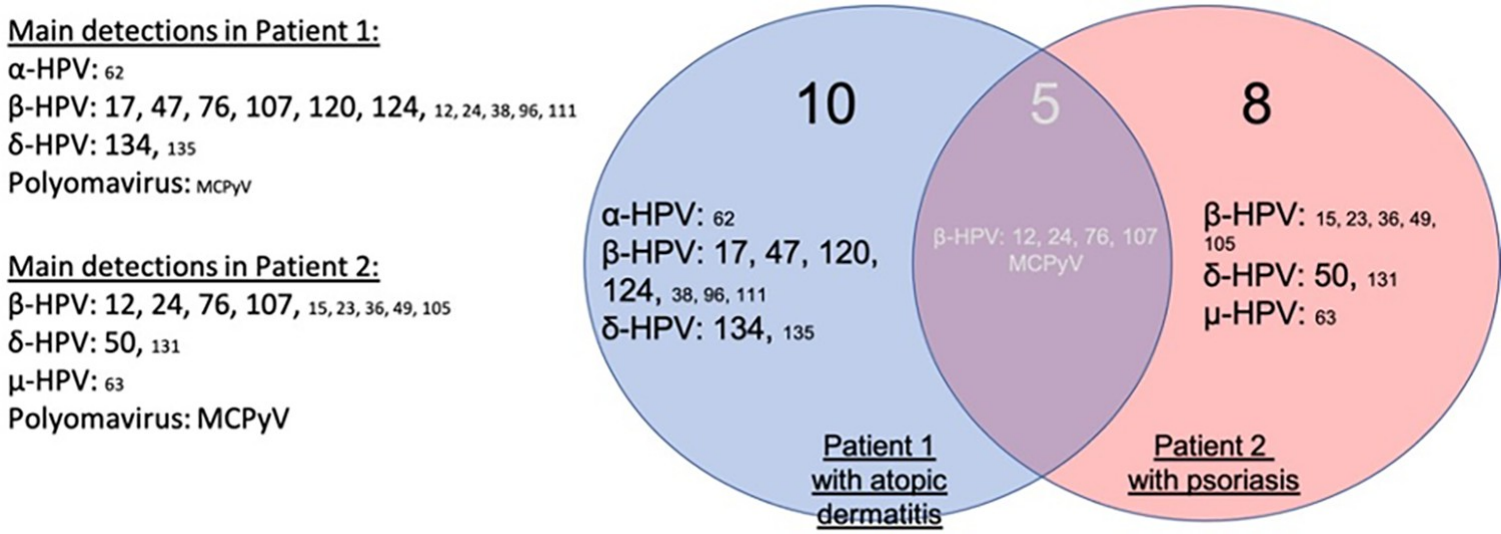
Venn diagram representing the main human papillomaviruses (HPV) and polyomaviruses (HPyV) detected in both patients. Left panel: List of HPVs and HPyVs strains detected in patient 1 and patient 2 individually. HPVs and HPyVs strains found in at least one sample (i.e. lesional or non-lesional skin; M0, M1 or M3) are indicated. Big letters: strains representing more than 10% of the skin virome. Small letters: strains representing more than 1% but less than 10% of the skin virome. Strains representing less than 1% of the skin virome are not depicted. Right panel: Specific and shared HPVs and HPyVs strains in both patients. The size of the letters does not apply to the intersection of the Venn diagram (grey).

The HPV and HPyV flora were analyzed with a AmpliSeq-HTS approach based on a custom AmpliSeq panel. As a general observation, high variability of the virome composition over time was observed for lesional sites, which contrasted with individual and stable signature for non-lesional skin. Overall, 31 and 32 types of HPVs and/or HPyVs species were detected in patients AD and PSO, respectively (S1 and S2 Tables), with some viruses being detected only at very low levels (<1%). Only 15 (patient AD) and 13 strains (patient PSO) accounted for more than 1% of the detected skin virome, among which only two beta-HPVs, HPV107 and HPV12, were found in common and represented a minor fraction (Fig 1).

By contrast, one specific gamma-HPV appeared to predominate in each patient, namely HPV 134 for patient AD (65% at M0; 93.1% at M1; 35% at M3) and HPV50 for patient PSO (88.3% at M0; 54.6% at M1; 92.6% at M3) (Table 1).

**Table 1:** First three over represented viruses at various time points in both patients.

Patient 1 with atopic dermatitis								
			NLS			LS		
Time of sampling			M0	M1	M3	M0	M1	M3
Nb of detected viruses (>1%)			3	3	10	7	7	6
Shared strains	M0/M1		2			4		
	M1/M3			3			5	
	M0/M1/M3		2			3		
First 3 over represented viruses (%)		1	**gHPV134 (64.97)**	**gHPV134 (93.08)**	**gHPV134 (35.07)**	bHPV107 (43.8)	bHPV107 (70.95)	bHPV120 (64.34)
		2	bHPV17 (33.72)	bHPV107 (3.27)	bHPV107 (18.87)	bHPV47 (42.02)	bHPV76 (12.26)	bHPV107 (10.31)
		3	bHPV107 (1.08)	bHPV96 (2.23)	bHPV124 (18.5)	bHPV17 (4.59)	aHPV62 (4.74)	bHPV47 (9.15)
Patient 2 with psoriasis								
			NLS			LS		
Time of sampling			M0	M1	M3	M0	M1	M3
Nb of detected viruses (>1%)			2	6	4	8	8	5
Shared strains	M0/M1		2			7		
	M1/M3			2			3	
	M0/M1/M3		1			3		
First 3 over represented viruses (%)		1	**gHPV50 (88.25)**	**gHPV50 (54.61)**	**gHPV50 (92.64)**	bHPV12 (56.69)	MCPyV (34.93)	MCPyV (37.79)
		2	bHPV76 (8.95)	bHPV76 (31.83)	bHPV105 (2.61)	bHPV24 (9.43)	bHPV12 (34.5)	bHPV107 (28.2)
		3	/	bHPV12 (6.44)	MuHPV63 (1.68)	gHPV131 (8.88)	bHPV76 (11.22)	bHPV24 (22.31)

NLS non-lesional skin, LS lesional skin, M month, MCPyV Merkel cell polyomavirus, HPV human papillomavirus.

In lesional skin (LS), among viruses accounting for more than 1% in at least one sample from M0 to M3 (*i*.*e*. 11 viruses for patient AD and 11 viruses for patient PSO; S1 and S2 Tables), beta-HPVs for patient AD (HPV107, 47, 17, 76, 124, 96, 24, 111, 120) and beta-HPVs (HPV12, 76, 24, 15, 107, 23, 36) together with MCPyV for patient PSO accounted for the majority of strains (95.76% at M0, 93.47% at M1 and 97.83% at M3 for patient AD; 80.63% at M0, 96.57% at M1 and 95.50% at M3 for patient PSO) (Table 1). In details, HPV 107 predominated at M0 and M1 (representing up to 70.95% of the viral population at M1) but HPV120 was the major component of the viral population at M3 (64.34% vs 10.31% for HPV107) in LS from patient AD; HPV12 predominated at M0 (56.69%) and remained predominant together with MCPyV at M1 (34.50% vs 34.93%), but its detection was strikingly reduced at M3 (consistently with both qPCR and NGS), while MCPyV (37.79%) and beta-HPVs other than HPV12 (28.20% for HPV107; 22.31% for HPV24) predominated at M3 in LS from patient PSO. Gamma-HPVs 134 and 50, which consistently predominated in NLS (up to 93.08% and 92.64% respectively) from patient AD and PSO respectively, represented less than 5% in any LS samples. Of note, in PSO LS treated with super potent topical steroids, the AmpliSeq RPM showed a decline for all the virus under scope over time. For instance, HPV12 was almost undetected at M3, whereas it was still detected in PSO NLS at M3.

### Correlation with qPCR

**Fig 2:**
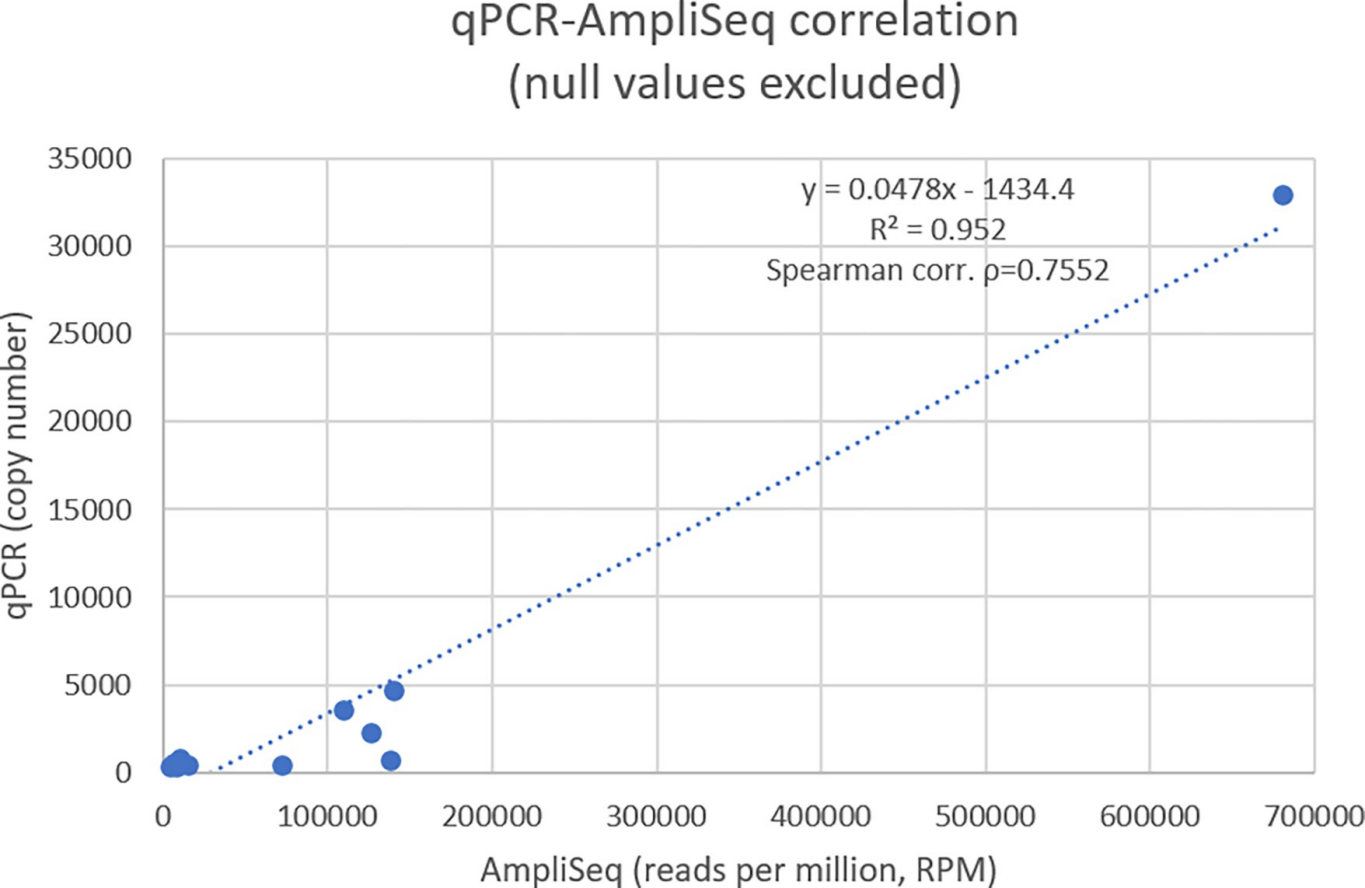
Correlation between AmpliSeq-HTS and qPCR (null values excluded). HTS high throughput sequencing, qPCR quantitative polymerase chain reaction.

In order to validate the quantitative analysis, we performed monoplex qPCR with specific primers for HPV107, 50, 134, 120, 12, and MCPyV on several but not all samples because of paucity of left DNA material. In total, 17 monoplex qPCRs were done in duplicates (S3 Table). Correlation between qPCR copy numbers and AmpliSeq-HTS read counts normalized by million reads trimmed (RPM) was high, with R^2^ = 0.95 and Spearman correlation ρ = 0.75 (Tables 2 and 3 and Fig 2). Importantly, qPCR detected and further quantified the corresponding viral loads whenever the number of detected reads with AmpliSeq-HTS reached at least 5,166 RPM (Tables 2 and 3), which indicates that AmpliSeq-HTS can be more sensitive than qPCR and is an appropriate technique to evaluate and compare actual viral loads in skin samples.

**Table 2:** Comparison of qPCR copy number and PCR/HTS reads per million (RPM) in lesional and non lesional skin samples at month 0, 1 and 3 (M0, M1, M3) from the patient with atopic dermatitis.

	Atopic dermatitis, lesional skin (antecubital fossae)						Atopic dermatitis, non lesional skin (inguinal folds)					
	M0		M1		M3		M0		M1		M3	
	qPCR copy number	AmpliSeq RPM	qPCR copy number	AmpliSeq RPM	qPCR copy number	AmpliSeq RPM	qPCR copy number	AmpliSeq RPM	qPCR copy number	AmpliSeq RPM	qPCR copy number	AmpliSeq RPM
HPV120-1	n.d.	0	n.d.	1	515	5166	n.d.	0	n.d.	47	n.d.	44
HPV120-2	n.d.		n.d.		34		n.d.		n.d.		n.d.	
HPV12-1	n.d.	3	n.d.	0	n.d.	0	n.d.	3	n.d.	0	n.d.	25
HPV12-2	n.d.		n.d.		n.d.		n.d.		n.d.		n.d.	
HPV107-1	145	9201	130	6955	n.d.	828	0	154	0	569	n.d.	273
HPV107-2	615		686		n.d.		0		0		n.d.	
HPV134-1	n.d.	356	n.d.	1	n.d.	0	26	9281	26	16181	0	507
HPV134-2	n.d.		n.d.		n.d.		413		623		0	
HPV50-1	n.d.	2	n.d.	0	n.d.	0	n.d.	1	n.d.	0	n.d.	3
HPV50-2	n.d.		n.d.		n.d.		n.d.		n.d.		n.d.	
MCPyV-1	n.d.	132	n.d.	0	n.d.	0	n.d.	3	n.d.	0	n.d.	20
MCPyV-2	n.d.		n.d.		n.d.		n.d.		n.d.		n.d.	

Each qPCR was done in duplicate ("-1" and "-2"). M months, n.d. no data

**Table 3:** Comparison of qPCR copy number and PCR/HTS reads per million (RPM) in lesional and non lesional skin samples at month 0, 1 and 3 (M0, M1, M3) from the patient with psoriasis.

	Psoriasis, lesional skin (elbows)						Psoriasis, non lesional skin (inguinal folds)					
	M0		M1		M3		M0		M1		M3	
	qPCR copy number	AmpliSeq RPM	qPCR copy number	AmpliSeq RPM	qPCR copy number	AmpliSeq RPM	qPCR copy number	AmpliSeq RPM	qPCR copy number	AmpliSeq RPM	qPCR copy number	AmpliSeq RPM
HPV120-1	n.d.	0	n.d.	0	n.d.	0	n.d.	0	n.d.	0	n.d.	0
HPV120-2	n.d.		n.d.		n.d.		n.d.		n.d.		n.d.	
HPV12-1	419	72972	736	139203	2	2	n.d.	1020	n.d.	13025	n.d.	1225
HPV12-2	564		502		4		n.d.		n.d.		n.d.	
HPV107-1	n.d.	1796	n.d.	13875	0	1405	n.d.	731	n.d.	2965	n.d.	12194
HPV107-2	n.d.		n.d.		0		n.d.		n.d.		n.d.	
HPV134-1	n.d.	0	n.d.	0	n.d.	0	n.d.	0	n.d.	0	n.d.	0
HPV134-2	n.d.		n.d.		n.d.		n.d.		n.d.		n.d.	
HPV50-1	n.d.	1268	n.d.	415	n.d.	217	3400	126764	4960	110528	65500	680982
HPV50-2	n.d.		n.d.		n.d.		960		2080		111	
MCPyV-1	1440	11204	5590	140961	0	1884	n.d.	677	n.d.	217	n.d.	1056
MCPyV-2	8		3540		0		n.d.		n.d.		n.d.	

Each qPCR was done in duplicate ("-1" and "-2"). M months, n.d. no data

## Discussion

AmpliSeq is now a widely used, commercially available NGS solution for both clinical and research laboratories. While the community of AmpliSeq users is on the rise, we developed this custom panel to help advance the study of the human skin virome in health and diseases.

With this high throughput sequencing using AmpliSeq targeting HPVs and HPyVs, we could show that at least 27 types of HPVs and/or HPyVs can be detected from 2 anatomical sites on the skin surface of two patients with AD and PSO. Interestingly, only 2 or 3 HPV types or HPyV species predominate, representing 72.44 to 99.77% of the detected HPVs and HPyVs. According to previous reports [15], beta- and gamma-HPVs were predominant in NLS, inguinal folds of both patients. In this work, we didn’t aim to compare the skin virome of the 2 patients but rather to evaluate the alterations of the skin virome over time in each individual. Hannigan et al. [5] showed that the skin virome was highly variable over time, unlike the gut virome [16]: at 4 weeks’ interval, less than 50% of the qualitative composition of the skin virome of a given body site in an individual behaved like commensal permanent resident, while the whole microbiome in the skin kept stable. However, viruses, although a minor component of the global biomass on skin surface, show individual and consistent signature along time. Thus, the predominance of HPV134 in patient AD and HPV50 in patient PSO in their NLS maintained from M0 to M3. On another hand in LS, resident viruses were replaced over time by a different and more variable population: the initial predominance of HPV107 in patient AD and of HPV12 in patient PSO faded over time, which may reflect a clinical improvement after topical or systemic treatment, with an over-representation at M3 of HPV120 in patient AD and of MCPyV in patient PSO.

In patient PSO, the number of viral types/species in NLS and in LS was similar. This result is not in line with previous studies showing that a higher number of beta-HPVs types are detected in psoriatic skin lesions than in healthy skin and plucked eyebrow hair [17], suggesting a more permissive immune system for beta-HPV in these patients [18]. Such differences could be linked to the method used for the quantification.

Alterations of the skin virome may result from an immunocompromised status. Foulongne et al. found a greater diversity of the skin virobiota in NLS swab sample from a patient diagnosed with Merkel Cell carcinoma [3], harboring the highest number of HPV reads, and being the only individual out of 6 harboring HPyV7 sequences. Pastrana et al. described a huge number of known (and new) gamma-papillomaviruses in genetically immunocompromised patients, commonly shed by healthy individuals but associated with florid warts in these patients [8]. Tirosh et al. [7] recently reported that eukaryotic viruses were more diverse and abundant in the skin of 27 genetically immunocompromised patients with DOCK8 deficiency (belonging to the spectrum of hyper-IgE syndromes) compared with 5 healthy individuals. On the contrary, bacterial and fungal communities’ composition and abundance were similar in the skin of these patients and the controls. Thus, the introduction of low-dose MTX, an immunosuppressive agent in patient AD, seemed to increase the diversity of HPVs (though gamma-HPV134 remained predominant) and/or HPyVs strains in NLS samples from 3 at M0 to 10 at M3, but not in LS samples (6 at M3 versus 7 at M0). This discrepancy in NLS versus LS samples could be explained by a greater impact of MTX on NLS flora because of an admitted increased excretion of MTX in folds.

On the contrary, in patient PSO receiving no systemic treatment, the predominance of HPV50 in NLS samples remained stable between M0 (88.25%) and M3 (92.64%). Also in this patient, the use of STS on LS was associated with a shift in the predominating viral strains. However, although efficient on PSO symptoms, the use of STS on LS did not increase the number of detected viruses, and did not restore a viral signature close to the NLS, unlike previous reports about STS influence on skin microbiota during flares of AD [19]. However, NLS and LS samples were taken from different anatomical sites, lowering the possibility to compare their composition. Additionally, the topography of the lesions in patient PSO were elbows, which are more often exposed to the environment and probably more susceptible for variation in their global skin microbiome over time.

## Conclusion

This approach combining multiplex PCR coupled with HTS is original and allows not only the detection but also the quantification of targeted viral communities. The technique correlated well with qPCR, being even more sensitive. Thus, it could be an interesting tool to promote studies of the cutaneous HPV and HPyV flora: contrarily to random NGS, and closer to what is widely used for bacteria with 16S RNA, it gives access to absolute abundance of each virus.

This work confirmed that the composition of the cutaneous HPV and HPyV commensal flora is variable between individuals and across body sites, but seems consistent over time in non-lesional skin only. The cutaneous HPV and HPyV flora was more diverse in lesional skin and seemed impacted by the activity of the treated inflammatory dermatitis over time. These results should be extended to larger cohorts and sampling areas, to give more insight regarding the sources of variability of the skin virome.

## Supporting information

S1 Fig Correlation between AmpliSeq-HTS and qPCR based on the 17 monoplex qPCRs that have been performed. a: qPCR null values were included. b: qPCR null values were excluded. HTS high throughput sequencing, qPCR quantitative polymerase chain reaction.
(TIF)

S1 Table Results obtained with AmpliSeq-HTS in lesional (antecubital fossae) and non-lesional (inguinal folds) skin samples from patient 1 with atopic dermatitis. Left panel: raw reads number (viruses with 0 read detected in all 6 conditions are not reported). Middle panel: reads numbers normalized by the total number of reads mapped to the amplicon list (%). Right panel: reads numbers normalized by million reads trimmed. HTS high throughput sequencing, qPCR quantitative polymerase chain reaction, RPM reads numbers normalized by million reads trimmed.
(XLSX)

S2 Table Results obtained with AmpliSeq-HTS in lesional (elbows) and non-lesional (inguinal folds) skin samples from patient 2 with psoriasis. Left panel: raw reads number (viruses with 0 read detected in all 6 conditions are not reported). Middle panel: reads numbers normalized by the total number of reads mapped to the amplicon list (%). Right panel: reads numbers normalized by million reads trimmed. HTS high throughput sequencing, qPCR quantitative polymerase chain reaction, RPM reads numbers normalized by million reads trimmed.
(XLSX)

S3 Table Amplicons sequences used to produce the plasmids for quantification of the viral loads in skin samples using qPCR. qPCR quantitative polymerase chain reaction.
(XLSX)
