# Supplementary figures and images for: A quantitative assay for the assessment of cutaneous human papillomaviruses and polyomaviruses over time: A proof-of-concept in two patients with atopic dermatitis and psoriasis
Source: PLoS One. 2024 Apr 3;19(4):e0297907. doi: 10.1371/journal.pone.0297907 (PMC10990162; doi:10.1371/journal.pone.0297907)

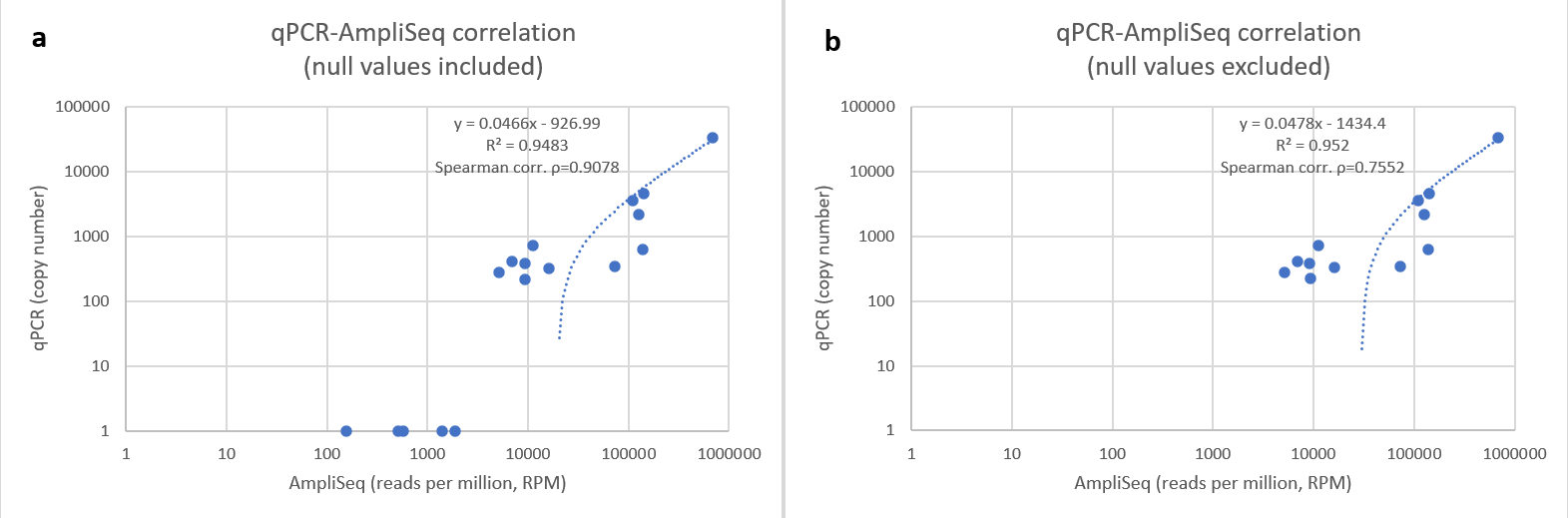

Supplement: S1 Fig — a: qPCR null values were included. b: qPCR null values were excluded. HTS high throughput sequencing, qPCR quantitative polymerase chain reaction. (TIF) [file pone.0297907.s001.tif]
